# Construction of genetic classification model for coronary atherosclerosis heart disease using three machine learning methods

**DOI:** 10.1186/s12872-022-02481-4

**Published:** 2022-02-12

**Authors:** Wenjuan Peng, Yuan Sun, Ling Zhang

**Affiliations:** Department of Epidemiology and Health Statistics, School of Public Health, Capital Medical University, and Beijing Municipal Key Laboratory of Clinical Epidemiology, No. 10, Xi Toutiao You Anmenwai, Fengtai District, Beijing, 100069 China

**Keywords:** Coronary atherosclerosis heart disease, Classification model, Machine learning, Support vector machine, Random forest, Logistic regression

## Abstract

**Background:**

Although the diagnostic method for coronary atherosclerosis heart disease (CAD) is constantly innovated, CAD in the early stage is still missed diagnosis for the absence of any symptoms. The gene expression levels varied during disease development; therefore, a classifier based on gene expression might contribute to CAD diagnosis. This study aimed to construct genetic classification models for CAD using gene expression data, which may provide new insight into the understanding of its pathogenesis.

**Methods:**

All statistical analysis was completed by R 3.4.4 software. Three raw gene expression datasets (GSE12288, GSE7638 and GSE66360) related to CAD were downloaded from the Gene Expression Omnibus database and included for analysis. Limma package was performed to identify differentially expressed genes (DEGs) between CAD samples and healthy controls. The WGCNA package was conducted to recognize CAD-related gene modules and hub genes, followed by recursive feature elimination analysis to select the optimal features genes (OFGs). The genetic classification models were established using support vector machine (SVM), random forest (RF) and logistic regression (LR), respectively. Further validation and receiver operating characteristic (ROC) curve analysis were conducted to evaluate the classification performance.

**Results:**

In total, 374 DEGs, eight gene modules, 33 hub genes and 12 OFGs (*HTR4*, *KISS1*, *CA12*, *CAMK2B*, *KLK2*, *DDC*, *CNGB1*, *DERL1*, *BCL6*, *LILRA2*, *HCK*, *MTF2*) were identified. ROC curve analysis showed that the accuracy of SVM, RF and LR were 75.58%, 63.57% and 63.95% in validation; with area under the curve of 0.813 (95% confidence interval, 95% CI 0.761–0.866, *P* < 0.0001), 0.727 (95% CI 0.665–0.788, *P* < 0.0001) and 0.783 (95% CI 0.725–0.841, *P* < 0.0001), respectively.

**Conclusions:**

In conclusion, this study found 12 gene signatures involved in the pathogenic mechanism of CAD. Among the CAD classifiers constructed by three machine learning methods, the SVM model has the best performance.

**Supplementary Information:**

The online version contains supplementary material available at 10.1186/s12872-022-02481-4.

## Background

Coronary atherosclerosis heart disease (CAD) is the most common cardiovascular diseases (CVDs) and is characterized by high morbidity and mortality [1]. CVD accounted for one-third of all deaths, and there were an estimated 17.92 million deaths due to CVDs worldwide in 2015 [2]. In China, the summary of China cardiovascular disease report (2018) estimated that about 290 million people are suffering from CVDs, and 11 million of them are CAD patients [3]. A previous study showed that over 40% of deaths in China are directly caused by CAD or its complications [4]. Therefore, a comprehensive analysis of multiple biomarkers interaction is of great significance to understand the pathogenesis of CAD.

With the development of technology, the diagnosis of CAD is constantly innovated. Invasive coronary angiography is so far the gold standard by which the presence and severity of CAD could be defined, especially in patients with significant left ventricular dysfunction [5]. Coronary computed tomography angiography is increasingly being considered as an alternative diagnostic method because of its effectiveness, safety and non-invasion [6]. In addition, magnetic resonance coronary angiography provides a superior soft tissue characterization, and is well suited to the detection of adverse plaque characteristics [7]. However, CAD in the early stage is still missed diagnosis for the absence of any symptoms or mild degree of disease [8]. CAD is influenced by both environmental [9, 10] and genetic factors [11]. Actually, gene expression levels varied before morphological abnormality of the tissue during CAD development [12]. Therefore, genetic classification models might contribute to CAD diagnosis.

Due to the extensive application of gene chip and next-generation sequencing technology, a large amount of gene expression data is stored in databases, for example, Gene Expression Omnibus (GEO, https://www.ncbi.nlm.nih.gov/geo/) [13]. GEO supplies plentiful data for researchers to investigate the association between gene expression and CAD [14–16]. Some analytical methods have been used as approaches for microarray data mining. The bioinformatics analyses could reveal the biological functions of CAD-related genes [17]. The machine learning methods contribute to finding genetic biomarkers or constructing classifiers of CAD [18].

In the present study, we obtained CAD-related gene chip data from GEO open resources. Differentially expressed genes (DEGs) were screened between CAD samples and healthy controls, followed by the weighted gene co-expression network analysis (WGCNA) [19] by which hub genes with the highest correlation with CAD were identified. Subsequently, the recursive feature elimination (RFE) [20] algorithm was performed to select the optimal features genes (OFGs) for CAD from hub genes. By utilizing machine learning methods, including support vector machine (SVM) [21], random forest (RF) [22] and logistic regression (LR), the genetic classification models of CAD were finally established. This study aimed to identify potential hub genes and construct genetic classification models for CAD, which may provide new insight into the understanding of its pathogenesis and facilitate further therapeutic studies.

## Materials

### Data collection, quality evaluation and preprocessing

In this study, three raw datasets (GSE12288, GSE7638 and GSE66360) and corresponding annotation files were acquired from GEO. The simpleaffy package was used to evaluate the quality of chips and draw a quality control diagram, the unqualified samples would be marked with “bioB” in the quality control diagram and further excluded. Then, affy package was performed to standardize raw data, including background correction, normalization, perfect match (PM) probe correction and probe expression value calculation. After that, robust multi-array average (RMA) algorithm [23] was conducted to normalize microarray data and perform a log2 transformation. The probe expression value is estimated based on a stochastic model employed by the PM signal distribution. Afterwards, each probe set in these three datasets was annotated with gene symbol according to corresponding annotation files. Furthermore, k-nearest neighbor (KNN) function in the impute package was carried out to fill in the missing data. Finally, the complete gene expression profiles were acquired. The impute.knn is a function to impute missing expression data using KNN [24]. For each gene with missing values, *k* nearest neighbors are selected using a Euclidean metric, and the missing elements are imputed by averaging those elements of its neighbors.

### Batch effect removal and differential expression analysis

The SVA package was carried out for correcting the batch effects of these three normalized datasets. The limma package [25, 26] was performed to identify DEGs between CAD samples and healthy controls in three datasets and the integrated dataset, respectively. And the Benjamini–Hochberg method was performed for multiple testing correction, by which the adjusted *P* value was calculated. The integrated dataset was the combination of GSE12288, GSE7638 and GSE66360. The thresholds of adjusted *P* < 0.001, |log_2_(foldchange, FC)|> 0.263 were set to define DEGs. Furthermore, volcano plots were achieved using ggplot2 package to investigate the whole gene comparison results.

### Weighted gene co-expression network analysis

Within the integrated dataset, the WGCNA package was conducted to construct the scale-free co-expression network and to identify hub genes from adjusted *P* < 0.001 genes. The theory behind WGCNA algorithm have been described in detail previously [27]. Firstly, the absolute value of correlation coefficient between the pair of genes i and j across of all subjects was defined as co-expression similarity (${\text{S}}_{{{\text{ij}}}} = \left| {{\text{cor}}\left( {{\text{i}},{\text{ j}}} \right)} \right|$). Therefore, ${\text{S}} = \left[ {{\text{S}}_{{{\text{ij}}}} } \right]$ was used to represent the co-expression correlation matrix. Secondly, the ${\text{S}}$ was transformed into an adjacency matrix by a power function: ${\text{a}}_{{{\text{ij}}}} = {\text{power}}\left( {{\text{S}}_{{{\text{ij}}}} ,\upbeta } \right) = \left| {{\text{S}}_{{{\text{ij}}}} } \right|^{\upbeta }$, where the soft thresholding power parameter, *β*, was set to 5 in this study. Thirdly, the topological overlap matrix (TOM) was calculated on the following function: ${\text{w}}_{{{\text{ij}}}} = \frac{{{\text{l}}_{{{\text{ij}}}} + {\text{a}}_{{{\text{ij}}}} }}{{{\text{min}}\left\{ {{\text{k}}_{{\text{i}}} ,{\text{k}}_{{\text{j}}} } \right\} + 1 - {\text{a}}_{{{\text{ij}}}} }}$, where ${\text{l}}_{{{\text{ij}}}} = \mathop \sum \limits_{\upmu } {\text{a}}_{{{\text{i}}\upmu }} {\text{a}}_{{{\text{j}}\upmu }}$, ${\text{k}}_{{\text{i}}} = \mathop \sum \limits_{{\upmu }} {\text{a}}_{{{{\text{i}\upmu }}}}$, ${\text{k}}_{{\text{j}}} = \mathop \sum \limits_{{\upmu }} {\text{a}}_{{{\text{i}\upmu }}}$. The μ denotes genes connected with gene i or j. Then, the dissimilarity was defined as ${\text{d}}_{{{\text{ij}}}}^{{\text{w}}} = 1 - {\text{w}}_{{{\text{ij}}}}$, thus forming a dissimilarity matrix. Finally, average linkage hierarchical clustering was conducted based on the TOM-based dissimilarity with a minimum size of 30 for the genes dendrogram to classify genes with similar expression profiles into modules. Each module was assigned to the corresponding color.

A dynamic hybrid branch cutting method was implemented on the TOM-based dendrogram to identify module eigengenes (ME). ME was calculated by the first principal component of a given module, which could represent the expression patterns of all genes. A phenotypic trait-based gene significance measure was defined as the absolute value of correlation between the gene i and the phenotypic trait (T): ${\text{GS}}_{{\text{i}}} = \left| {{\text{cor}}\left( {{\text{i}},{\text{T}}} \right)} \right|$. T is the binary variable for CAD status (patient status = 1 and healthy control = 0). GS_i_ denotes the association between gene i and T. Module membership (MM) represent the correlation between gene i and ME: ${\text{MM}}_{{\text{i}}} = \left| {{\text{cor}}\left( {{\text{i}},{\text{ME}}} \right)} \right|$, which explains associations between gene i and the corresponding module. Hub genes represent a series of genes that is significantly connected to a relevant module [28]. In the current study, a cut of $|{\text{GS}}_{{\text{i}}} | > 0.2$, $\left| {{\text{MM}}_{{\text{i}}} } \right| > 0.8$ was considered as the threshold of hub genes.

### Selection of optimal feature gene sets

RFE was applied to select OFGs of CAD from hub genes in the integrated dataset using caret package. The OFGs can be used as identifiers of clinical diagnosis to construct a CAD classifier based on their expression levels. Performances of different types of samples were evaluated through combinations of iterative random features until the optimal feature combination was obtained. And, the number of cross-validation was set to 200 in this study. Later, the heatmap of the OFGs was drawn by pheatmap package to compare the expression levels between groups in datasets, respectively.

### Construction and validation of genetic classification models

Three machine learning methods (SVM, RF and LR) were used to construct the CAD genetic classification models. SVM is a discriminant classifier defined by the classification hyperplane. The model is trained with labeled training samples, and then, the test samples are classified by the output of the optimal hyperplane [29]. RF is an integrated learning algorithm that combines different decision trees. Among the decision trees that constitute an RF model, each tree is an independent set generated based on random samples. Each tree learns and predicts independently, and the final result is determined by the mean value of all decision trees [30, 31]. LR is one of the GLM models, which have been regarded as an extension of the linear model that establishes the relationship between the mathematical expected value of the response variables and the predictive variables of the linear combination through the coupling function [32].

In the present study, 50% of samples in GSE12288 were selected randomly and used as a training dataset. The SVM, RF and LR classification models were constructed using e1071 package, randomForest package and glm function, respectively. Samples were classified into cases and controls according to the expression level of genes. To confirm the robustness and transferability of these constructed classifiers, internal and external validations were performed. Internal validation was carried out in the remaining 50% of samples of GSE12288, and external validation was performed in the combination of GSE7638 and GSE66360 datasets. Then, the efficacy of models was comprehensively evaluated in terms of sensitivity (Se), specificity (Sp), positive predictive value (PPV), negative predictive value (NPV) and the area under the ROC curve (AUC). All statistical analyses were conducted using R 3.4.4 software.

## Results

### Data information

**Fig. 1:**
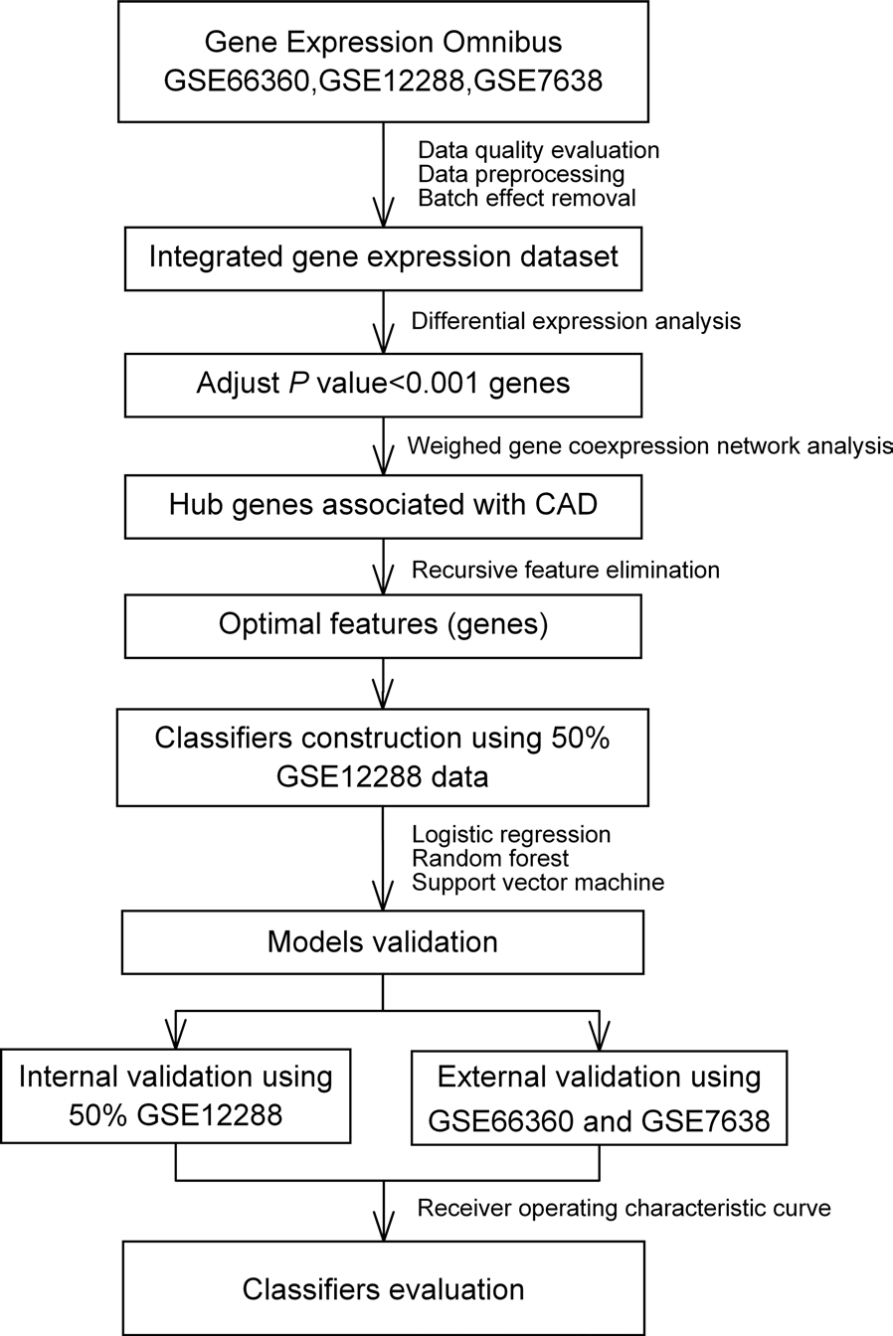
Schematic overview of study flow. CAD, coronary atherosclerosis heart disease

Figure 1 summarized the schematic overview of the study flow. In this study, three gene expression datasets related to CAD were acquired. The information of them was summarized in Table 1. A total of 481 samples were included for analysis, among which 269 samples were CAD patients and 212 were healthy control samples. After evaluation of chip quality, one sample (GSM1620893) belonging to healthy control group in GSE66360 was dropped (Additional file 1: Figure S1).

**Table 1:** Information of the downloaded datasets

Dataset	Case/control	Country	Specimen	Probe number	Platform
GSE12288	110/112	Switzerland	Peripheral blood	22483	GPL96
GSE66360	49/50	USA	Circulating endothelial cells	47000	GPL570
GSE7638	110/50	Switzerland	Peripheral monocyte	14500	GPL571

### Integration of three datasets and identification of differentially expressed genes

**Fig. 2:**
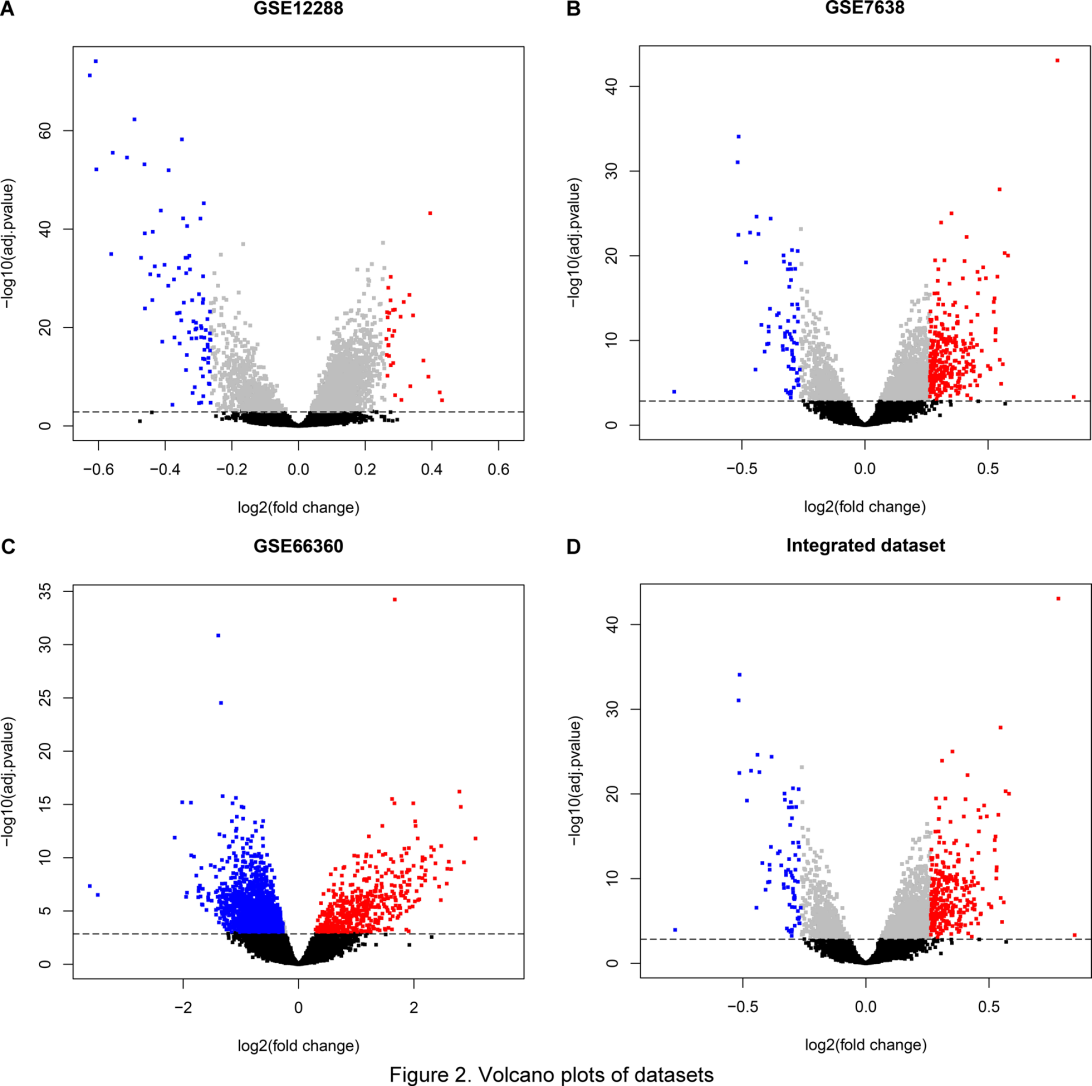
Volcano plots of datasets. The red nodes represent genes that adjusted *P* < 0.001 and log_2_FC > 0.263. The blue nodes represent genes that adjusted *P* < 0.001 and log_2_FC < − 0.263. The horizontal dotted line represents adjusted *P* = 0.001. Integrated dataset was the combination of GSE12288, GSE7638 and GSE66360. The analysis of differentially expressed genes between case group and control group was performed using Limma package

After batch effect removal analysis, 12,395 genes and 480 samples remained in the integrated dataset. DEGs were identified by differential expression analysis (Fig. 2). Briefly, when CAD samples were compared with healthy controls, 114 (31 upregulated and 83 downregulated), 1157 (1112 upregulated and 45 downregulated) and 2484 (471 upregulated and 2013 downregulated) DEGs were recognized in GSE12288, GSE7638 and GSE66360, respectively (Fig. 2A–C). And, 374 DEGs were identified in the integrated dataset (Fig. 2D) in which 303 DEGs were upregulated and 71 were downregulated.

### Hub genes identification using WGCNA

**Fig. 3:**
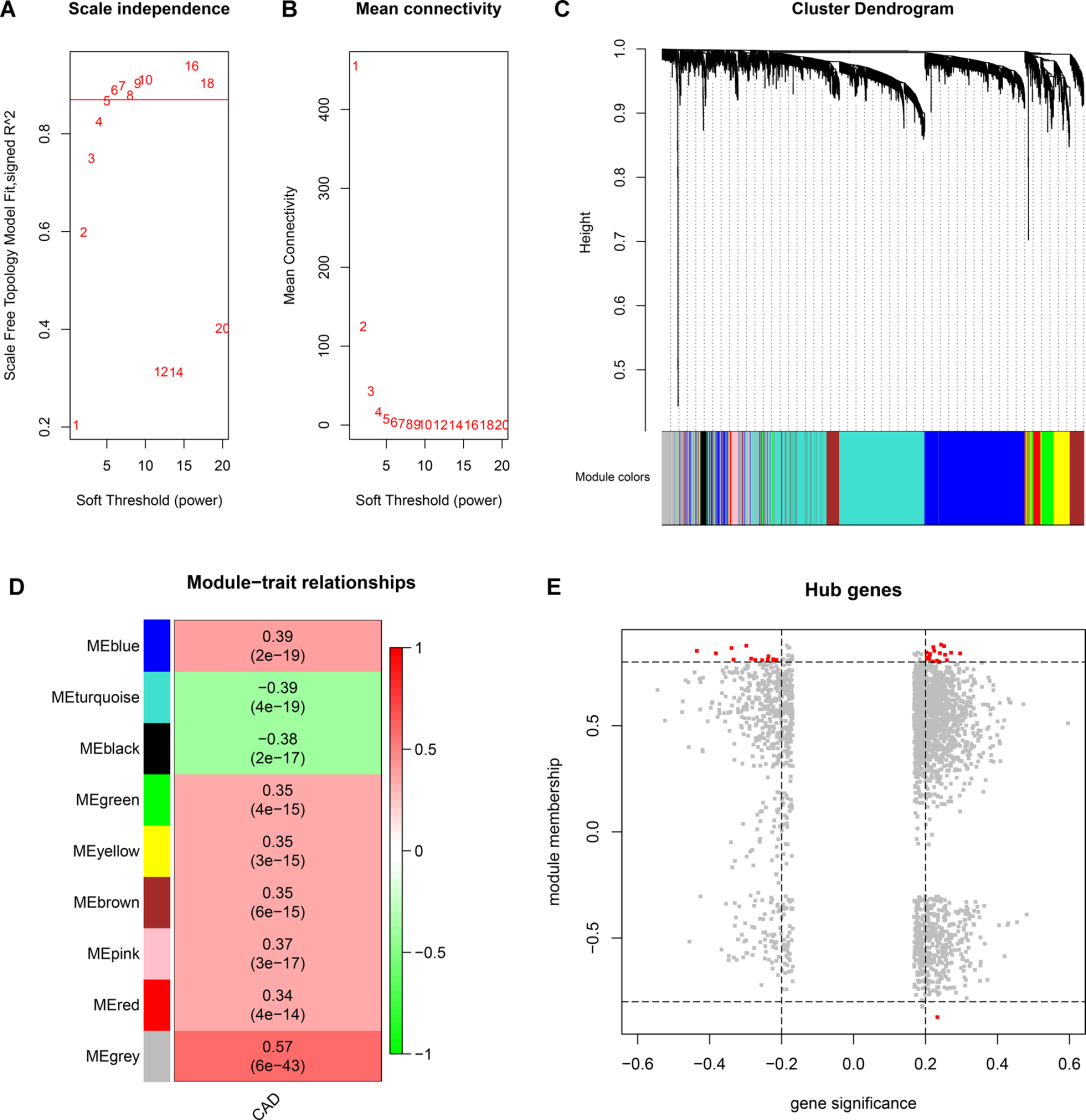
Weighted gene co-expression network analysis. **A, B** Scale-free network test by which the soft thresholding power parameter was set to 5. **C** Hierarchical clustering. The branches of the tree represent the clusters of genes. The colors below the tree were gene modules that correspond to the clusters. **D** The correlation between gene modules and traits (disease), and red represents a positive correlation and green represents a negative correlation. **E** Hub genes. The red nodes represent hub genes screened by the threshold of absolute gene significance > 0.2 and absolute module membership > 0.8. The vertical dotted line represents absolute gene significance = 0.2, and the horizontal dotted line represents absolute module membership = 0.8. CAD, coronary atherosclerosis heart disease

The expression data of 2546 genes that adjusted *P* < 0.001 were analyzed using WGCNA package to identify the co-expression patterns and hub genes. The threshold power of *β* = 5 was selected to ensure a scale‐free network (Fig. 3A, B). The co-expression network contained eight modules in total and the module sizes ranged from 40 (pink) to 859 (turquoise). These modules were labelled with colours and depicted in the dendrograms provided in Fig. 3C. However, 387 genes were not similarly co-expressed with other genes in the network (grey). The associations between the MEs of modules and CAD status (patient status = 1 and healthy control = 0) were identified (Fig. 3D). The correlation coefficients (*r*) of modules indicated that they were all significantly correlated with CAD status (*P* < 0.05). The MEs of blue, green, yellow, brown, pink and red modules were positively correlated with CAD status (*r* > 0, *P* < 0.05), while MEs of turquoise and black modules were negatively correlated with CAD status (*r* < 0, *P* < 0.05). In this study, $|{\text{GS}}_{{\text{i}}} | > 0.2$ and $\left| {{\text{MM}}_{{\text{i}}} } \right| > 0.8$ was considered as threshold for identifying hub genes, and 33 genes were identified from six modules in total (Fig. 3E, Table 2).

**Table 2:** The information of 33 hub genes identified by weighed gene co-expression network analysis

Gene symbol	Module	GS	*P*.GS	MM	*P*.MM
*ZAP70*	Black	− 0.273	1.11E−09	0.810	8.54E−113
*HTR4*	Blue	0.224	6.76E−07	0.853	6.29E−137
*CA12*	Blue	0.203	7.61E−06	0.845	7.38E−132
*KLK2*	Blue	0.209	4.07E−06	0.821	3.04E−118
*DERL1*	Brown	0.232	2.79E−07	0.807	3.38E−111
*NFIL3*	Green	0.211	3.01E−06	0.839	2.35E−128
*BCL6*	Green	0.240	1.03E−07	0.842	5.09E−130
*FPR1*	Green	0.220	1.09E−06	0.802	4.99E−109
*ACSL1*	Green	0.213	2.63E−06	0.834	2.15E−125
*CSF3R*	Green	0.210	3.35E−06	0.815	3.28E−115
*C5AR1*	Green	0.238	1.35E−07	0.801	2.41E−108
*NCF2*	Green	0.271	1.58E−09	0.844	2.57E−131
*CNGB1*	Turquoise	− 0.298	2.63E−11	0.877	4.70E−154
*DDC*	Turquoise	− 0.334	5.92E−14	0.811	1.47E−113
*CAMK2B*	Turquoise	− 0.339	2.17E−14	0.866	9.86E−146
*HCN2*	Turquoise	− 0.222	8.74E−07	0.808	5.01E−112
*MUC13*	Turquoise	− 0.436	1.21E−23	0.852	1.03E−136
*KISS1*	Turquoise	− 0.383	3.52E−18	0.841	1.55E−129
*JPH2*	Turquoise	− 0.254	1.64E−08	0.808	4.63E−112
*ADRA2C*	Turquoise	− 0.214	2.16E−06	0.810	9.52E−113
*MTF2*	Turquoise	0.233	2.49E−07	− 0.872	9.16E−151
*LHX5*	Turquoise	− 0.237	1.55E−07	0.827	8.63E−122
*EMID1*	Turquoise	− 0.239	1.23E−07	0.811	3.21E−113
*HSD17B14*	Turquoise	− 0.285	2.14E−10	0.816	7.91E−116
*RTEL1*	Turquoise	− 0.223	7.75E−07	0.813	3.04E−114
*PRKCD*	Yellow	0.296	3.58E−11	0.841	2.05E−129
*LILRA2*	Yellow	0.254	1.56E−08	0.834	1.42E−125
*PILRA*	Yellow	0.222	9.12E−07	0.870	3.90E−149
*PGD*	Yellow	0.259	8.04E−09	0.808	4.25E−112
*APLP2*	Yellow	0.205	5.73E−06	0.816	7.83E−116
*LYN*	Yellow	0.203	7.19E−06	0.822	4.14E−119
*HCK*	Yellow	0.243	6.66E−08	0.882	3.17E−158
*TYROBP*	Yellow	0.252	2.25E−08	0.875	2.03E−152

GS, gene significance with coronary atherosclerosis heart disease; *P*.GS, *P* value for gene significance with coronary atherosclerosis heart disease; MM, module membership; *P*.MM, *P* value for module membership

### Construction of genetic classification models based on optimal feature genes

**Fig. 4:**
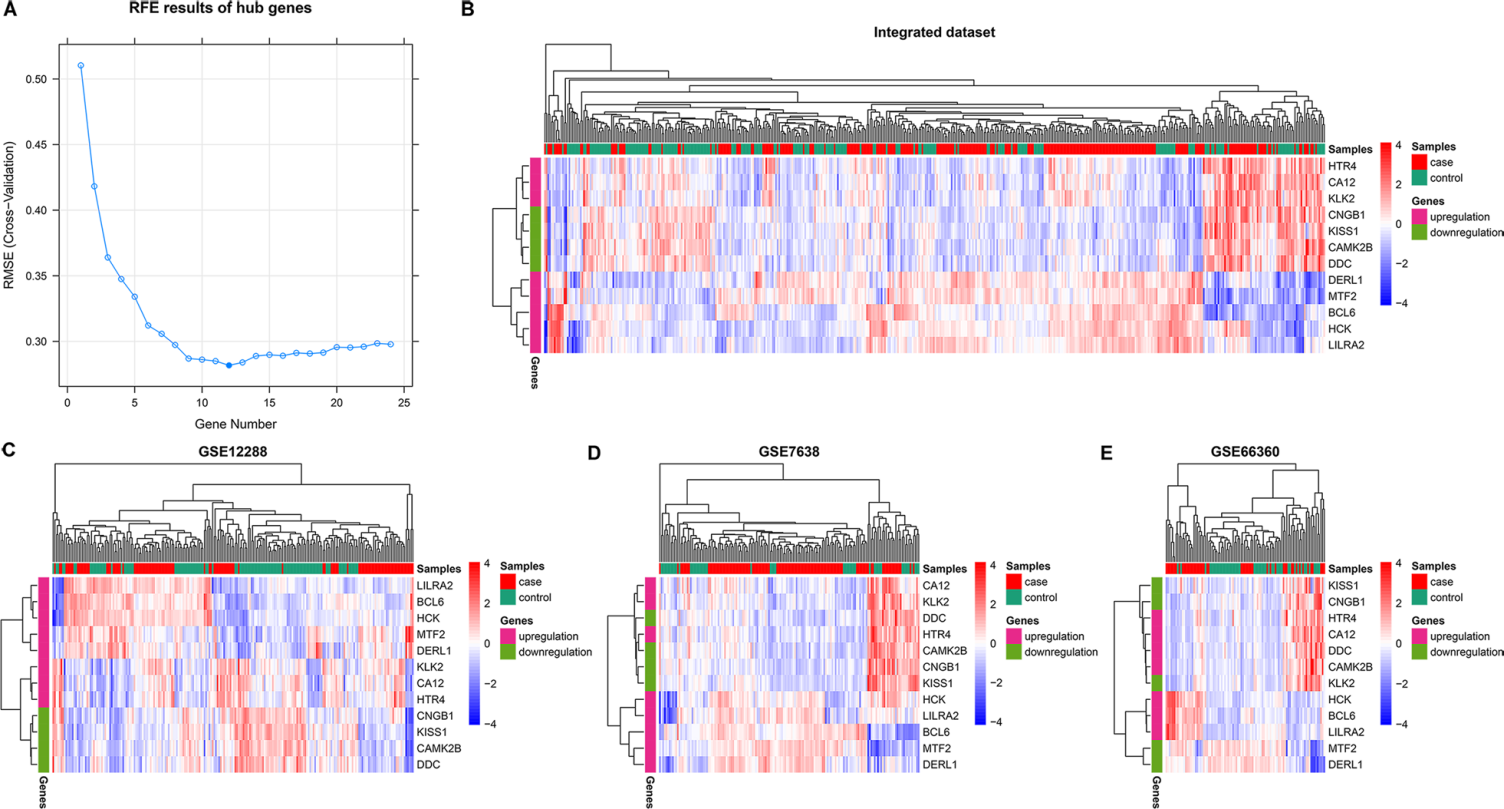
Feature elimination curves of hub genes and heatmap of the 12 optimal feature genes in different dataset. **A** Feature elimination curves of hub genes. Root mean square error (RMSE) is the statistical parameter to determine the optimal feature genes after the analysis of recursive feature elimination algorithm. The lowest RMSE correspond with the best optimal feature gene set, based on which the model was trained by machine learning methods in 50% samples in GSE12288. **B–E** Heatmap of the 12 optimal feature genes in different dataset using pheatmap package. The red and blue colors indicate high and low expression, respectively, of the 12 optimal feature genes among samples. Upregulation, genes that higher expressed in case group than control group. Downregulation, genes that lower expressed in case group than control group. Integrated data was the combination of GSE12288, GSE7638 and GSE66360

In order to obtain the optimal characteristic combination of genes representative of 33 hub genes, the RFE algorithm was adopted in the integrated dataset. Finally, 12 hub genes were selected as OFGs, this OFGs combination had the lowest classification root mean square error (RMSE) of 25.54% (Fig. 4A). These 12 OFGs were *HTR4*, *KISS1*, *CA12*, *CAMK2B*, *KLK2*, *DDC*, *CNGB1*, *DERL1*, *BCL6*, *LILRA2*, *HCK* and *MTF2* (Table 3), among which eight OFGs (*HTR4*, *CA12*, *KLK2*, *DERL1*, *BCL6*, *LILRA2*, *HCK* and *MTF2*) were upregulated, while the other four (*KISS1*, *CAMK2B*, *CNGB1* and *DDC*) were downregulated. Hierarchical clustering analysis was then carried out in datasets based on expression data of OFGs (Fig. 4B–E).

**Table 3:** The result information of 12 optimal feature genes in limma package analysis

Gene symbol	GSE12288		GSE7638		GSE66360		Integrated dataset	
	Foldchange	Adjusted *p*	Foldchange	Adjusted *p*	Foldchange	Adjusted *p*	Foldchange	Adjusted *p*
*BCL6*	1.07	2.36E−01	1.20	7.07E−03	2.86	2.93E−09	1.27	1.34E−06
*CA12*	1.05	4.18E−04	1.06	1.99E−03	1.00	9.94E−01	1.08	6.80E−05
*CAMK2B*	0.88	1.02E−22	0.95	4.57E−02	1.02	8.29E−01	0.90	3.94E−12
*CNGB1*	0.89	2.72E−08	0.93	1.39E−02	0.83	4.21E−02	0.88	1.22E−09
*DDC*	0.90	1.03E−17	0.92	6.26E−03	1.00	9.87E−01	0.88	7.23E−12
*DERL1*	1.03	1.91E−01	1.29	4.81E−19	0.88	4.74E−01	1.17	3.29E−06
*HCK*	1.07	1.67E−01	1.16	5.43E−06	2.09	1.10E−03	1.28	9.15E−07
*HTR4*	1.11	4.11E−10	1.00	9.68E−01	1.09	4.12E−01	1.10	8.22E−06
*KISS1*	0.78	3.49E−22	0.90	3.42E−02	0.92	2.26E−01	0.84	1.05E−15
*KLK2*	1.08	5.39E−09	1.06	3.58E−02	0.88	1.69E−01	1.09	3.93E−05
*LILRA2*	1.04	3.88E−01	1.19	1.74E−06	2.22	7.91E−06	1.25	2.60E−07
*MTF2*	1.07	1.17E−04	1.27	1.02E−08	0.81	1.22E−01	1.15	3.07E−06

Integrated dataset was the combination of GSE12288, GSE7638 and GSE66360; foldchange, the fold change of the average gene expressional level going from control group to case group; adjusted *P*, the *P* value adjusted by Benjamini–Hochberg in comparing the gene expressional level between case group and control group

In the current study, the training dataset contained 111 (50%) samples in GSE12288 which were selected randomly. The SVM, RF and LR classifiers were constructed based on the expression of these 12 genes in the training dataset. Furthermore, the remained 50% of samples of GSE12288, and the combination of GSE7638 and GSE66360 were deem to testing datasets for internal and external validation, respectively.

### Validation and evaluation of classifiers performance

**Fig. 5:**
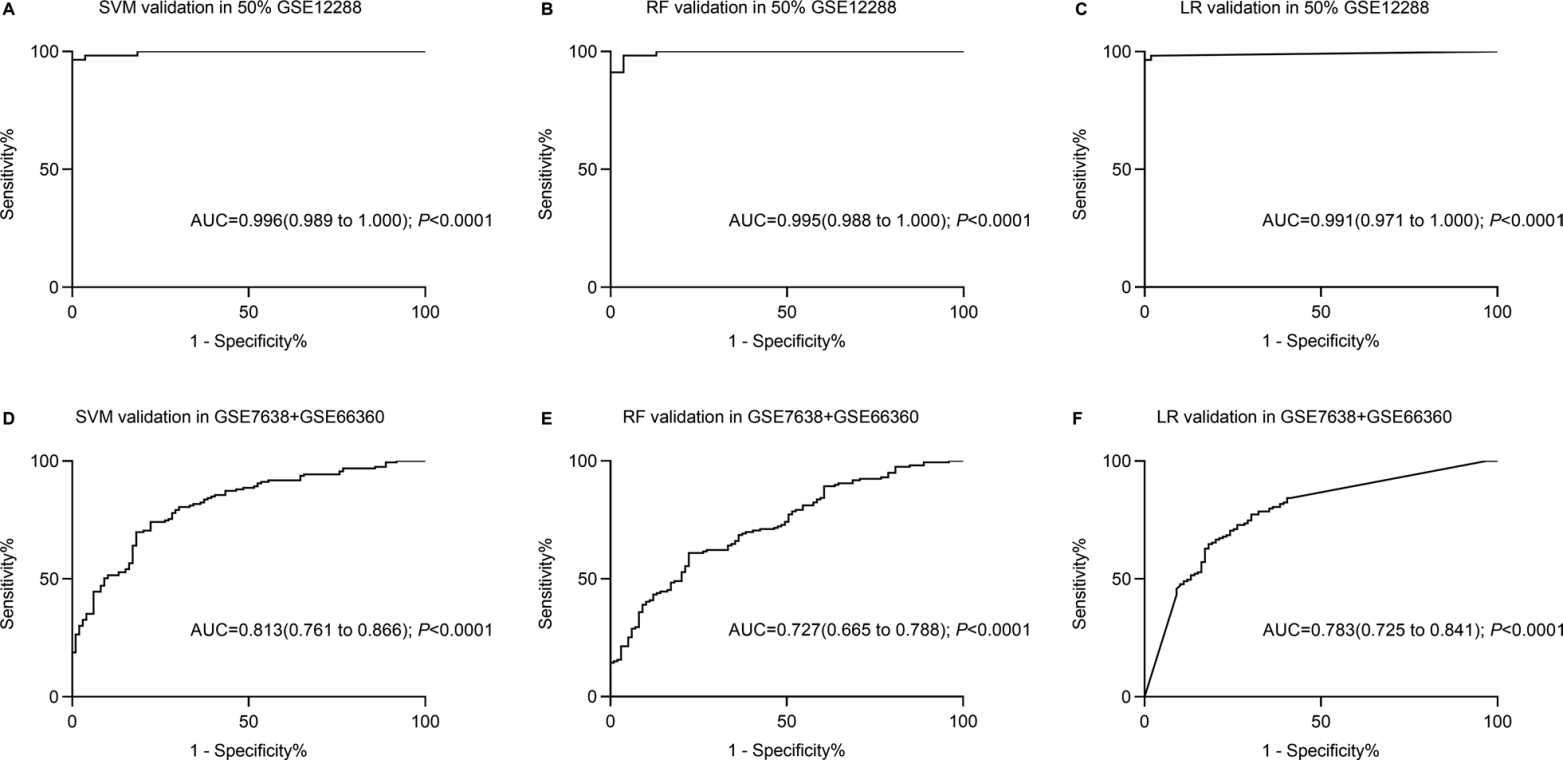
ROC charts of classification by SVM, RF and LR classifiers in internal and external validation datasets. SVM, support vector machine; RF, randomforest; LR, logistic regression; AUC, area under the ROC curve; ROC, receiver operating characteristic curve

The results showed that SVM, RF and LR classifiers could accurately classify 105 (94.59%), 106 (95.50%) and 108 (97.30%) of the 111 samples in internal validation, respectively. In external validations, 195 (75.59%) of the 258 samples were accurately classified via SVM classifier, with AUC of 0.813 (95% confidence interval (95% CI): 0.761–0.866, *P* < 0.0001). RF classifier could exactly category 164 (63.57%) of 258 samples, with AUC of 0.727 (95% CI 0.665–0.788, *P* < 0.0001). LR classifier could precisely classify 165 (63.95%) of 258 samples, with AUC of 0.783 (95% CI 0.725–0.841, *P* < 0.0001). The ROC charts of samples were shown in Fig. 5.

The performance of these three classifiers was evaluated using a variety of indicators, such as correct rate, Se, Sp, PPV and NPV, which were described in Table 4. In the internal validation, the accuracy appeared RF > LR > SVM, but the AUC SVM > RF > LR. In the external validation, both correct rate and AUC appeared SVM > LR > RF. The Se in SVM classifier was the highest (0.780, 95% CI 0.707–0.842) and in LR classifier was the lowest (0.516, 95% CI 0.435–0.596), respectively. The Sp in LR classifier was the highest (0.869, 95% CI 0.786–0.928) and in RF classifier was the lowest (0.525, 95% CI 0.422–0.627). These results suggested that the constructed SVM classifier based on the 12 OFGs could be the best in the present study.

**Table 4:** Validation and evaluation results of three machine learning classifiers performance

Classifiers	AUC (95% CI)	Se (95% CI)	Sp (95% CI)	PPV	NPV	Correct rate
SVM ^a^	0.996 (0.989, 1.000)	0.982 (0.906, 1.000)	0.907 (0.797, 0.969)	0.918	0.946	0.946
SVM ^b^	0.813 (0.761, 0.866)	0.780 (0.707, 0.842)	0.717 (0.618, 0.803)	0.816	0.756	0.756
RF ^a^	0.995 (0.988, 1.000)	0.983 (0.906, 1.000)	0.907 (0.797, 0.969)	0.919	0.955	0.955
RF ^b^	0.727 (0.665, 0.788)	0.723 (0.647, 0.791)	0.525 (0.422, 0.627)	0.696	0.636	0.636
LR ^a^	0.991 (0.971, 1.000)	0.965 (0.879, 0.996)	0.982 (0.901, 1.000)	0.982	0.973	0.973
LR ^b^	0.783 (0.725, 0.841)	0.516 (0.435, 0.596)	0.869 (0.786, 0.928)	0.859	0.640	0.640

SVM, support vector machine; RF, randomforest; LR, logistic regression; Se, sensitivity; Sp, specificity; PPV, positive predictive value; NPV, negative predictive value; AUC, area under the ROC curve; ROC, receiver operating characteristic curve

^a^Verified in the 50% samples of GSE12288 (111/222)

^b^Verified in the integrated dataset of GSE7638 and GSE66360 (258)

## Discussion

Based on gene expression data, machine learning methods can be applied in constructing classification models of disease and propose a deeper understanding for clinical diagnosis and treatment. In this study, three mRNA expression profiles related to 269 CAD and 212 healthy control samples were downloaded from GEO. A total of 374 DEGs and 33 hub genes were identified by bioinformatics analyses. Accordingly, 12 OFGs (*HTR4*, *KISS1*, *CA12*, *CAMK2B*, *KLK2*, *DDC*, *CNGB1*, *DERL1*, *BCL6*, *LILRA2*, *HCK* and *MTF2*) were obtained and classification models were constructed through three machine learning methods. Finally, results of evaluating classifiers performance showed the SVM model was the best in the present study, with the AUC of 0.813 (95% CI 0.761–0.866), the sensitivity of 0.780 (95% CI 0.707–0.842) and the specificity of 0.717 (95% CI 0.618–0.803), respectively.

Gene expression might change before morphological abnormality of the tissue, researchers demonstrated that macrophage C-type lectin receptor CLEC5A (MDL-1) mainly expressed in atherosclerotic lesional macrophages and elevated macrophage MDL-1 expression was associated with early plaque progression [12]. Pulanco MC et al. found that C1q promoted macrophage survival and improved foam cell function, which may play an important protective role in early atherosclerosis progression [33]. In addition, matrix metalloproteinases (MMPs) participated in different mechanisms fundamental to atherothrombotic progression [34, 35], such as MMP-12 [36] and MMP-2 [37].

In the current study, eight (*HTR4*, *CA12*, *KLK2*, *DERL1*, *BCL6*, *LILRA2*, *HCK* and *MTF2*) of 12 potential critical genes were upregulated. Oksala found that *CA12* expression was elevated in atherosclerotic plaques compared to control tissues (internal thoracic artery controls). And CA12 protein was expressed in the atheromatous core and to some extent in all vessel layers in plaques of all vessel beds, while only sparse cells were positive in control vessels [38]. Chronic inflammation is a hallmark of atherosclerosis, Barish GD examined the impact of the transcriptional repressor BCL6 on atherogenesis and revealed BCL6-SMRT/NCoR complexes could constrain immune responses and contribute to the prevention of atherosclerosis [39]. HCK and FGR are two Src tyrosine kinases, Medina demonstrated that Hck/Fgr-deficiency leads to reduced atherosclerotic lesion with concomitant reductions in macrophage accumulation and, paradoxically, lesion stability [40]. HTR4 is a member of the family of serotonin receptors and associated with average and maximal carotid intima-media thickness measures [41]. Serotonin, also named as 5-hydroxytryptamine (5-HT), is a well-known vasoreactive amine that could affect the circulation of the heart. Human kallikrein 2 (KLK2, also called hK2) has an important in vivo regulatory function on Prostate-specific antigen (PSA) activity, and could convert the inactive precursor form of PSA to active PSA [42]. PSA is a member of the human kallikrein family of serine proteases [43] and PSA is an established marker of myocardial infarction [44].

The other four (*KISS1*, *CAMK2B*, *CNGB1* and *DDC*) of 12 OFGs were downregulated. The encoded protein of DDC catalyzes the decarboxylation of L-5-hydroxytryptophan to serotonin, which is a well-known vasoreactive amine. Kisspeptins are the endogenous cleavage products of the KiSS1 protein, they function as potent vasoconstrictors, and the response could comparable to angiotensin (Ang)-II in the coronary artery; In addition, Kisspeptins’ receptor, G protein-coupled receptor 54, is discretely located at atherosclerosis-prone vessels [45]. The product of *CAMK2B* belongs to serine/threonine protein kinase family. Akt (a serine/threonine protein kinase B) is an important signaling mediator which includes various Akt isoforms, such as Akt1, Akt2, and Akt3 [46]. Researchers reported that, in apoE-deficient mice, the loss of Akt1 leaded to severe atherosclerosis [47] and Akt3 deficiency in macrophages promoted foam cell formation and atherosclerosis [48]. T lymphocytes participate in the chronic inflammatory reaction and ultimately lead to the occurrence and development of acute coronary syndrome (ACS) [49, 50]. *CNGB1* also called *GARP*, Zhu et al*.* found that the expression of *GARP* in CD4^+^ T cells of ACS patients was lower than those of control patients [51]. Circulating CD4^+^ CD25^+^ GARP^+^ Tregs were impaired in patients with ACS, targeting GARP might promote the protective function of Tregs in ACS [52].

This study used three kinds of machine learning methods (SVM, RF and LR) to construct genetic classification model of CAD. The SVM, RF and LR have been widely applied for discriminant analyses or biomarker identification in diseases, such as acute coronary syndromes [53], osteosarcoma [54], lung adenocarcinoma [55], rheumatoid arthritis [56], chronic obstructive pulmonary disease [57]. Several studies also compared these three classifiers to find the best one as disease classification models [58–60]. In the present study, the SVM classifier showed the best classification efficacy (AUC in the internal and external validation were 0.996 and 0.813, respectively) and was considered as the optimal machine learning method in this study.

Some strengths and limitations of the current study should be acknowledged. Firstly, feature gene selection was the basis of the model construction, this study conducted both WGCNA and RFE algorithm to identify gene features. WGCNA is an advanced systems biology-based approach used for finding molecular mechanisms and for linking the information to phenotypic traits [19]. WGCNA has been widely and successfully used to identify candidate biomarkers and gene modules highly associated with disease [19]. The combined application of WGCNA and RFE in the current study might find the optimal gene features associated with CAD to the maximum extent. Secondly, we included a sufficient number of samples, excluded the unqualified sample, and removed the batch effect between datasets, which made our statistical analyses more reliable. Thirdly, this study performed three kinds of machine learning methods to construct classifiers, and the classification efficacy was compared. Finally, both internal and external validation were conducted to examine the performance of three classifiers and the best classifier was selected. Limitations were as follows: Firstly, we only analyzed the gene expression profiles, but the clinic information was not taken into account since the data was not available. Secondly, the optimal feature genes related to CAD should be further validated by real-time polymerase chain reaction with a larger sample size and functional experiments. Eventually, whether the genetic classification model could be used in practice is currently unknown and should be explored in future studies.

## Conclusions

In conclusion, 33 CAD-related hub genes were identified using bioinformatics analyses, and 12 OFGs were obtained. Among the CAD classifiers constructed by three machine learning methods, SVM model has the best performance, which proposed a deeper understanding for CAD clinical diagnosis and treatment.

## Supplementary Information

**Additional file 1: Figure S1**. Quality control diagram of GSE666360. The horizontal axis is the scale factor (SF), which refers to the average signal level of all probe in the chip. Each sample has two values, the first number represents the detection rate, which refers to the number of probes with signal divided by the number of all probes in the chip; the second number represents the background noise, which refers to the average signal level of all mismatch probes. The hollow triangle is actin3/actin5, and is marked red when the value greater than 3, marked blue when the value less than 3. The hollow circle is gapdh3/gapdh5, and is marked red when the value greater than 1.25, marked blue when the value less than 1.25. Solid circle with wire refers to SF of sample, and the sample (GSM1620893) marked with "bioB" is unqualified and further excluded.

## Data Availability

The datasets analyzed during the current study are available in the GEO (https://www.ncbi.nlm.nih.gov/geo/) databases.

## Abbreviations

CAD: Coronary atherosclerosis heart disease
CVD: Cardiovascular disease
DEG: Differentially expressed gene
WGCNA: Weighted gene co-expression network analysis
RFE: Recursive feature elimination
OFG: Optimal features gene
SVM: Support vector machine
RF: Random forest
LR: Logistic regression
FC: Foldchange
Se: Sensitivity
Sp: Specificity
PPV: Positive predictive value
NPV: Negative predictive value
ROC: Receiver operator characteristic
AUC: Area under the ROC curve
